# Beyond Biomarkers: Blending Copeptin and Clinical Cues to Distinguish Central Diabetes Insipidus from Primary Polydipsia in Children

**DOI:** 10.3390/biomedicines13102573

**Published:** 2025-10-21

**Authors:** Diana-Andreea Ciortea, Carmen Loredana Petrea (Cliveți), Gabriela Isabela Verga (Răuță), Sorin Ion Berbece, Gabriela Gurău, Silvia Fotea, Mădălina Nicoleta Matei

**Affiliations:** 1 Faculty of Medicine and Pharmacy, “Dunarea de Jos” University of Galati, 800008 Galati, Romania; diana.ciortea@ugal.ro (D.-A.C.); carmen.petrea@ugal.ro (C.L.P.); gabriela.verga@ugal.ro (G.I.V.); gabriela.gurau@ugal.ro (G.G.);; 2 “Maria Sklodowska Curie” Emergency Clinical Hospital for Children, 041451 Bucharest, Romania; 3 ”Sf Ioan” Emergency Clinical Hospital for Children, 800487 Galati, Romania

**Keywords:** copeptin, diabetes insipidus, polydipsia, polyuria, child, biomarker, decision making, logistic models, regression analysis, clinical decision support

## Abstract

**Background**: Polyuria–polydipsia syndrome (PPS) in children poses a major diagnostic challenge, as central diabetes insipidus (CDI) and primary polydipsia (PP) require distinct treatments. Although copeptin is a robust diagnostic biomarker, using only fixed thresholds may not adequately support decision making in borderline cases. To address this gap, we evaluated a multimodal diagnostic approach that integrates copeptin dynamics with clinical profiling. **Methods**: In a prospective diagnostic study (2019–2025), 24 children with PPS (CDI = 11, PP = 13) underwent hypertonic saline testing with serial sodium, osmolality, and copeptin sampling. Predictors included stimulated copeptin, peak sodium, peak osmolality, test duration, and tolerability. A Ridge regression model was applied and internally validated with stratified cross-validation. **Results**: Stimulated copeptin was the strongest discriminator, while sodium/osmolality dynamics and tolerability provided complementary value. The multimodal model achieved cross-validated AUC of 0.937 with 83.3% accuracy, and the procedure was safe and feasible in children. These findings support moving beyond biomarker cut-offs toward integrative diagnostic approaches that better reflect real-world clinical practice. **Conclusions**: Combining copeptin with clinical profiling in a penalized regression framework yields a robust and interpretable tool for distinguishing CDI from PP. More broadly, such integrative models may enhance diagnostic precision in rare pediatric disorders and provide a foundation for future multicenter validation and clinical decision-support applications.

## 1. Introduction

Polyuria–polydipsia syndrome (PPS) in children remains a major diagnostic challenge, as its differential diagnosis includes central diabetes insipidus (CDI), nephrogenic diabetes insipidus (NDI), and primary polydipsia (PP). PPS is relatively rare in children but carries disproportionate clinical consequences. The reported prevalence of CDI is around 1 in 25,000 live births, while NDI occurs in approximately 4 in 1,000,000, and PP is more frequently encountered in adolescents, particularly in association with behavioral or psychiatric disorders [1,2,3,4,5].

Each entity has distinct pathophysiological mechanisms and requires different therapeutic approaches. In CDI, impaired or absent secretion of arginine vasopressin (AVP) from the posterior pituitary leads to an inability to concentrate urine, necessitating lifelong replacement with desmopressin to prevent dehydration and hypernatremia [6,7,8,9]. In NDI, renal resistance to AVP—most often due to X-linked AVPR2 mutations or autosomal aquaporin-2 defects—renders desmopressin ineffective, requiring alternative management such as thiazide diuretics, amiloride, and careful fluid balance [3,10,11,12,13]. In contrast, PP is a behavioral or hypothalamic disorder characterized by excessive fluid intake, where desmopressin treatment may provoke life-threatening hyponatremia and management instead relies on fluid restriction and addressing underlying causes [14,15]. These fundamental differences underline the clinical urgency of accurate and timely differentiation, since inappropriate treatment—such as unnecessary or delayed desmopressin administration—can lead to severe complications, including dehydration, hyponatremia, or permanent renal damage [15,16,17,18].

Despite being relatively uncommon, the consequences of misdiagnosis are substantial: patients may undergo unnecessary imaging, face repeated hospitalizations, or develop complications from inappropriate management [19]. These challenges situate PPS at the intersection of endocrinology, nephrology, and pediatric acute care, underscoring the urgent need for more robust, multimodal diagnostic strategies.

The conventional diagnostic pathway relies heavily on the water deprivation test (WDT), which is time-consuming, poorly tolerated in pediatric patients, inconclusive in borderline cases, and carries risks of dehydration and psychological distress [17,20,21]. Safer and more reliable alternatives are therefore urgently needed.

In this context, attention has shifted toward biomarkers such as copeptin, which offer a physiologically grounded alternative to functional testing. Copeptin, the C-terminal segment of the AVP precursor, has emerged as a robust and stable biomarker that reflects AVP secretion. At the cellular level, osmosensory neurons in the hypothalamus detect extracellular osmolality changes through mechanosensitive TRPV channels and aquaporin-associated signaling, which regulate AVP gene transcription and secretion [22,23,24,25,26]. These mechanisms provide the biological basis for using copeptin as a surrogate marker of AVP release in disorders of water balance. Its diagnostic role has been validated in several studies, particularly after osmotic stimulation with hypertonic saline infusion [27,28,29,30,31]. A stimulated copeptin cutoff of <6.5 pmol/L has been shown to differentiate CDI from PP with high sensitivity and specificity, while basal copeptin ≥ 21.4 pmol/L is strongly indicative of NDI [27,32,33,34,35,36].

Despite these advances, reliance on a single biomarker may not adequately capture interindividual variability or the full clinical context of PPS. Several factors complicate interpretation, including variability in baseline sodium levels, incomplete osmotic stimulation during testing, and patient-specific tolerance to the procedure [37]. Furthermore, in real-world pediatric cohorts, borderline copeptin values are not uncommon, creating uncertainty for clinicians. These limitations highlight persistent diagnostic gaps that neither traditional nor biomarker-based strategies fully resolve. To overcome these limitations, recent research has moved toward integrative strategies that combine biomarkers with clinical parameters.

Recent evidence suggests that diagnostic accuracy may be improved by integrating biomarker dynamics with clinical variables such as plasma sodium, serum osmolality, test duration, and patient tolerability [38]. These multimodal approaches mirror clinical reasoning more closely than single-parameter strategies and are increasingly used to construct composite diagnostic tools [39,40]. They require, however, statistical frameworks capable of handling small, correlated datasets, which are often typical of rare pediatric disorders.

At the methodological level, penalized regression with cross-validation provides a rigorous framework to reduce overfitting and enhance reproducibility in rare pediatric conditions. Traditional logistic regression is unstable in small cohorts, as is often the case in pediatrics. Penalized regression, particularly Ridge regression, adds a regularization term that shrinks coefficients toward zero, reducing variance without eliminating predictors altogether [41]. This approach maintains interpretability while improving predictive stability. Cross-validation, especially in stratified form, is recommended to provide unbiased estimates of model performance in small datasets [42]. These methodological strategies have been successfully applied in other rare pediatric disorders. For example, penalized regression, often considered a machine learning–based approach, has been successfully used in neurodegenerative disorders such as Friedreich ataxia, where composite biomarkers outperformed individual measures despite small cohorts [43]. Similarly, our team previously applied Ridge regression for predictive modeling in pediatric SIADH, integrating serum and urinary parameters related to AVP activity. That study demonstrated that penalized regression with internal cross-validation can improve diagnostic stratification in rare pediatric conditions [44]. Building on this methodological experience, we applied a similar framework to PPS, this time integrating stimulated copeptin with clinical parameters into a practical multimodal diagnostic algorithm.

Despite progress in biomarker validation, diagnostic uncertainty persists in pediatric PPS, particularly in cases where copeptin results fall close to established thresholds [45,46]. In addition, sodium and osmolality dynamics, as well as patient tolerability, are rarely incorporated into diagnostic algorithms, although they directly affect both accuracy and feasibility in clinical practice [47]. Finally, traditional statistical approaches often lack robustness in small pediatric cohorts, underscoring the need for penalized regression frameworks that can enhance stability while preserving interpretability.

These limitations underscore the need for innovative diagnostic frameworks and define the rationale for the present study.

Based on these premises, we hypothesized that combining stimulated copeptin with clinical profiling in a multimodal algorithm could improve the diagnostic performance for PPS in children. In this study, we developed and internally validated a Ridge logistic regression model with stratified cross-validation. Our aim was to demonstrate that an integrated algorithm, rather than a single cutoff value, can more effectively differentiate between CDI and PP, while also assessing feasibility and tolerability in the pediatric setting.

## 2. Materials and Methods

The methodological approach of this study was based on the dual need to ensure clinical relevance and statistical rigor in evaluating pediatric polyuria–polydipsia syndrome (PPS). Given the rarity of PPS in pediatrics, a prospective diagnostic accuracy study is particularly valuable, as it minimizes bias and enhances reproducibility [48]. Within this framework, the hypertonic saline infusion test was adopted because it can provide a rapid and controlled osmotic stimulus with well-documented safety and diagnostic accuracy for copeptin measurement [27,31,49]. Copeptin itself was selected as the biomarker of interest not only for its greater stability in plasma compared with arginine vasopressin (AVP), but also for its validated role as a reliable surrogate of AVP release [50,51,52]. At the statistical level, the small sample sizes typical of pediatric PPS necessitate robust modeling strategies. In order to address this particular context, penalized regression methods, such as Ridge regression, are increasingly recommended, as they limit overfitting, enhance stability, and maintain interpretability [53]. Taken together, these considerations guided the present study design, aiming a sound and reliable methodological approach.

### 2.1. Study Design and Setting

This study aimed to develop and validate a multimodal diagnostic model for pediatric PPS, integrating stimulated copeptin levels with clinical parameters. Data were derived from a prospective, cross-sectional diagnostic accuracy study conducted between 2019 and 2025 at the “Sf Ioan” Emergency Clinical Hospital for Children, Galati, Romania. The protocol followed the Standards for Reporting of Diagnostic Accuracy Studies (STARD) guidelines [54].

Importantly, this analysis is distinct from our previously published work, which focused on the comparative accuracy of water deprivation testing versus hypertonic saline–stimulated copeptin [36]. In contrast, the present study advances the field by developing and internally validating a multimodal Ridge regression model that integrates copeptin with clinical profiling, moving beyond biomarker thresholds toward algorithmic decision support.

### 2.2. Study Population and Inclusion Criteria

Children aged 2–17 years presenting with persistent hypotonic polyuria (>2.5 L/m^2^/day or >3 mL/kg/h) and polydipsia (>2 L/m^2^/day), with or without nocturia, were eligible for inclusion. Patients with confirmed nephrogenic diabetes insipidus (NDI) were excluded, as the study objective focused on distinguishing central diabetes insipidus (CDI) from primary polydipsia (PP).

From an initial cohort of 27 patients who underwent copeptin testing, 24 (14 males, 10 females) were included in this final analysis, and 3 children with genetically or clinically confirmed NDI were excluded to maintain homogeneity of the diagnostic model. Of the 24 patients included, 11 were diagnosed with CDI and 13 with PP. The CDI group included 4 males and 7 females, whereas the PP group included 8 males and 5 females. Detailed demographic and comparative data are further presented in Section 3.1.

Additional exclusion criteria were acute dehydration, chronic kidney disease, diabetes mellitus, use of medications influencing vasopressin release (e.g., corticosteroids, diuretics), and incomplete datasets.

The final reference diagnosis (CDI vs. PP) was established based on stimulated copeptin thresholds in conjunction with clinical follow-up. Patients with stimulated copeptin < 6.5 pmol/L and persistent symptoms were classified as CDI (complete or partial), while patients with copeptin > 6.5 pmol/L who demonstrated preserved thirst perception and spontaneous resolution of symptoms during follow-up were classified as PP.

Written informed consent was obtained from all guardians, including agreement for the use of anonymized data in scientific research. The study complied with the Declaration of Helsinki (2013 revision) and the General Data Protection Regulation (GDPR). Ethical approval was granted by the Medical Ethics Committee of the “Sf Ioan” Emergency Clinical Hospital for Children in Galati, Romania, (approval numbers: 6257/1 October 2019 and 7650/2 April 2025).

### 2.3. Diagnostic Protocol and Biomarker Testing

All patients underwent a standardized hypertonic saline infusion test (3% NaCl). The protocol consisted of an initial 250 mL bolus followed by continuous infusion at 0.15 mL/kg/min. Serum sodium and osmolality were monitored at regular intervals. The infusion was discontinued once serum sodium reached ≥150 mmol/L or serum osmolality ≥ 300 mOsm/kg [20,36]. This protocol was selected because it provides controlled osmotic stimulation of hypothalamic osmoreceptors, thereby inducing AVP and copeptin release in a physiologically reproducible manner, with a shorter duration and better tolerability than the traditional water deprivation test [17,27].

Venous blood samples for copeptin were collected (1) at baseline and (2) at the stimulation endpoint. Samples were processed under standardized pre-analytical conditions (EDTA tubes, immediate centrifugation at 4 °C, storage at −80 °C). Copeptin concentrations were measured using a standardized fluorescence immunoassay (FIA) on the BRAHMS LIA platform (Thermo Fisher Scientific, Hennigsdorf, Germany) [36].

The rest of the serum and urinary parameters were analyzed using the Ortho Vitros 4600 and Vitros FS 5.1. Chemistry Analyzer (Ortho Clinical Diagnostics, Raritan, NJ, USA), according to the manufacturer’s recommendation. For osmolality the following formula was used: Osmolality = 1.86 × [Na^+^] + (Glucose/18) + (BUN/2.8).

Test duration and subjective tolerability were recorded for each child. Tolerability was rated by the supervising clinician on a 3-point Likert scale (1 = good, 2 = moderate, 3 = poor). The test was well tolerated in all cases, with no serious adverse events. Minor limitations include interindividual variability in osmotic sensitivity and transient discomfort during infusion, which were mitigated through close monitoring and predefined safety stopping criteria.

### 2.4. Statistical Analysis

The primary objective was to construct and internally validate a predictive diagnostic model capable of differentiating CDI from PP. All statistical analyses were performed in Python Idle 3.11 (libraries: scikit-learn v1.4.2, pandas, matplotlib) and cross-checked in R (version 4.4.1, RStudio) for reproducibility.

The distribution of continuous variables was tested for normality using the Shapiro–Wilk test. As several parameters deviated from normality, continuous variables were summarized as medians with interquartile ranges (IQRs) and compared between groups using the Mann–Whitney U test. Categorical variables were compared using Fisher’s exact test. Associations between copeptin, sodium, osmolality, and age were examined using Spearman’s rho (ρ), chosen for its robustness in non-normally distributed biomedical data. Multicollinearity was assessed via variance inflation factors (VIFs), with a cutoff of 5.

**Figure A1:**
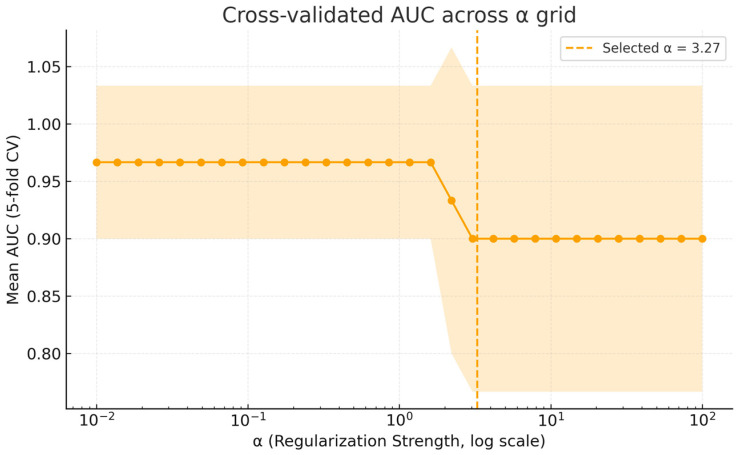
Cross-validated AUC across a broad α grid (10^−2^–10^2^, log scale). The Ridge model demonstrated stable performance with mean AUC consistently above 0.93 across the plateau range, confirming that the selected α = 3.27 falls within the optimal interval. The shaded band represents standard deviation across folds.

**Figure A2:**
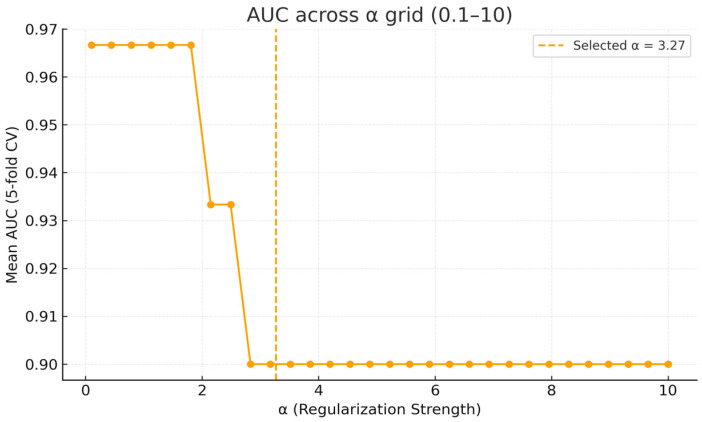
Cross-validated AUC across a narrower α grid (0.1–10). Zooming into the range used for LogisticRegressionCV highlights the same plateau, with minimal variation in AUC, reinforcing that diagnostic performance is insensitive to small perturbations in α.

**Figure A3:**
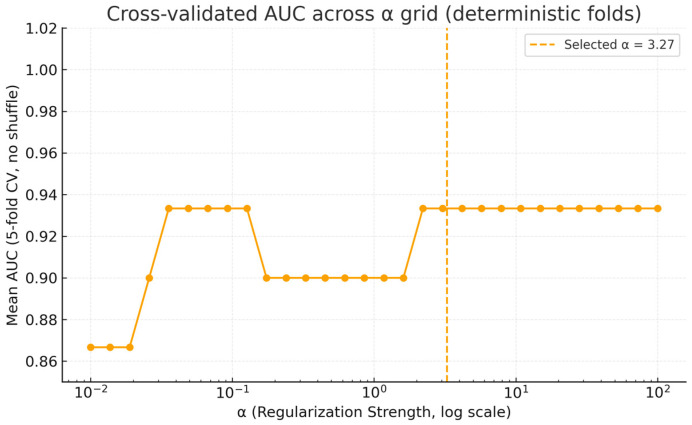
Cross-validated AUC across α grid using deterministic (non-shuffled) folds. Stability was maintained when class partitions were fixed rather than randomized, excluding the possibility that α selection was an artifact of random fold allocation.

For predictive modeling, we applied a Ridge logistic regression model (L2 regularization) to account for intercorrelations and reduce overfitting. All continuous predictors were standardized using z-scores prior to modeling. The regularization strength (α) was optimized across a predefined grid (0.001–100). Model calibration was guided by mean cross-validated area under the ROC curve (AUC), with sensitivity to the regularization strength (α) examined across a predefined grid. The final α (3.27) was chosen from the stable performance plateau, as documented in Appendix A (Figure A1, Figure A2 and Figure A3).

The outcome was binary (1 = CDI, 0 = PP). Baseline sodium, osmolality, and copeptin were excluded from the final model to ensure reliance on dynamic, stimulation-based parameters.

Model performance was assessed using sensitivity, specificity, accuracy, and AUC. Internal validation was performed using 5-fold stratified cross-validation, preserving class distribution across folds to reduce imbalance-related bias. Performance metrics were averaged across folds, and aggregated confusion matrices are reported. Regression coefficients, full-sample metrics, and cross-validation results are detailed in Section 3.

## 3. Results

### 3.1. Baseline Characteristics and Comparative Analysis

A total of 24 pediatric patients with polyuria–polydipsia syndrome were included in the final analysis, comprising 11 children with central diabetes insipidus (CDI) and 13 with primary polydipsia (PP). Three patients with genetically or clinically confirmed nephrogenic diabetes insipidus (NDI) from the original cohort of 27 were excluded to maintain homogeneity of the diagnostic model. Patients with partial CDI were classified within the CDI group, consistent with previously validated copeptin thresholds.

Continuous variables are presented as medians with interquartile ranges (IQRs). A detailed comparative summary of clinical and biochemical variables between CDI and PP groups is presented in Table 1. As shown in this table, the median age was significantly lower in CDI patients compared with PP, with corresponding differences in body weight. Given the wide age range (2–17 years), we tested whether age or body weight systematically influenced test duration or copeptin dynamics. However, no significant correlations were found between test duration and either age (ρ = 0.43, *p* = 0.15) or body weight (ρ = 0.16, *p* = 0.60), indicating that the diagnostic protocol was not systematically influenced by patient size or developmental stage.

Stimulated copeptin levels clearly discriminated between groups, while basal copeptin values were also significantly lower in CDI. In contrast, baseline and maximum serum sodium, as well as serum osmolality values, did not differ significantly between groups.

Regarding tolerability, CDI patients tended to report slightly worse scores compared to PP, although overall tolerability was acceptable across the cohort. Test duration was shorter in CDI compared with PP, but this difference did not reach statistical significance.

#### Correlation Analysis Between Copeptin Levels, Serum Parameters, and Test Tolerability

**Figure 1:**
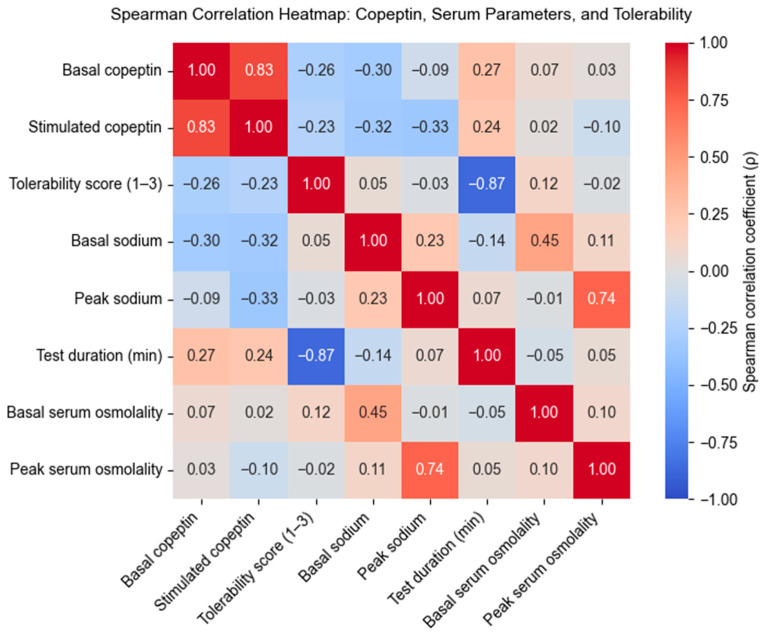
Spearman correlation heatmap—illustrating the interrelationships among basal and stimulated copeptin, serum sodium and osmolality (baseline and peak), test duration, and tolerability scores. The observed patterns highlight the integrated physiological response to osmotic challenge and the potential utility of combining biomarker and clinical parameters in diagnostic stratification.

Spearman correlation analysis was used to explore the interrelationships between copeptin concentrations, biochemical responses, and tolerability outcomes during the hypertonic saline infusion test. The overall correlation matrix is presented in Figure 1.

Basal and stimulated copeptin levels were strongly correlated (ρ = 0.83, *p* < 0.001), confirming the internal consistency of copeptin secretion across baseline and stimulated states. Stimulated copeptin showed a weak inverse association with both peak serum sodium (ρ = –0.33, *p* = 0.092) and baseline sodium (ρ = –0.32, *p* = 0.101), although these did not reach statistical significance. Physiological integrity was supported by a strong positive correlation between peak sodium and peak osmolality (ρ = 0.74, *p* < 0.001), as well as a moderate correlation between baseline sodium and baseline osmolality (ρ = 0.45, *p* = 0.020). Importantly, neither basal nor stimulated copeptin levels correlated significantly with tolerability scores, suggesting that subjective discomfort is not directly influenced by biomarker values.

Tolerability itself was strongly and inversely correlated with test duration (ρ = –0.87, *p* < 0.001), indicating that prolonged infusion was the main driver of patient discomfort.

Beyond numerical scores, clinical symptoms were systematically monitored during testing. Transient complaints such as headache, nausea, and shivering occurred in a minority of patients, but none required premature test discontinuation. No serious adverse events were observed. Tolerability scores were assigned by a single supervising pediatric specialist, which avoided interrater variability but also limited the ability to assess interobserver reliability. We acknowledge that the simplified 3-point Likert scale, although pragmatic in a pediatric setting, does not capture the full spectrum of symptom burden. Future studies could consider validated pediatric tolerability instruments for a more granular assessment.

Together, these findings highlight that while copeptin dynamics provide robust diagnostic separation, complementary clinical parameters—particularly tolerability and sodium–osmolality interactions—offer additional value to a multimodal diagnostic framework in pediatric PPS.

### 3.2. Multicollinearity Assessment and Predictor Selection

Prior to constructing the predictive model, we assessed multicollinearity among candidate predictors to ensure statistical robustness. Variance inflation factors (VIFs) were calculated for all continuous variables obtained from the hypertonic saline test (Table 2), including basal and stimulated copeptin concentrations, serum sodium (baseline and peak), serum osmolality (baseline and peak), test duration, and tolerability scores.

All predictors yielded VIF values below the conventional threshold of 5, indicating acceptable levels of interdependence. Basal and stimulated copeptin showed moderate collinearity (Table 2), consistent with their expected physiological coupling, but not reaching redundancy. Similarly, peak sodium and peak osmolality displayed modest correlation, reflecting osmotic dynamics, as shown in Table 2. These findings supported the simultaneous inclusion of biochemical and clinical parameters in the regression model.

Beyond statistical validity, the choice to include test-related parameters (duration and tolerability) was driven by clinical relevance. Stimulated copeptin has shown excellent diagnostic accuracy in controlled settings [12,17,23], but real-world implementation requires consideration of feasibility and patient burden. Importantly, in our previous work, discrepancies between the water deprivation test (WDT) and copeptin-based classification were observed: several patients initially categorized as CDI or inconclusive by WDT were reclassified as PP following copeptin testing and clinical follow-up [17]. Such discordance underscores the limitations of relying on a single test and highlights the need for integrative diagnostic frameworks. Test duration reflects resource utilization, while tolerability captures safety and acceptance—factors critical when comparing hypertonic saline infusion to conventional alternatives such as the WDT or arginine stimulation.

Although hypertonic saline–stimulated copeptin alone may reach near-perfect performance in selected cohorts, sole reliance on fixed thresholds does not fully address interindividual variability, borderline cases, or incomplete stimulation. Multimodal modeling allows the combination of complementary signals, improving robustness and maintaining diagnostic accuracy across broader clinical contexts. Finally, we acknowledge that additional clinical or molecular markers—such as validated thirst perception scales or genetic variants affecting AVP signaling—were not available in this study but may further enhance multimodal algorithms in future research.

### 3.3. Ridge Logistic Regression Modeling and Internal Validation

To evaluate the diagnostic utility of copeptin and associated clinical parameters, we constructed a penalized logistic regression model (L2-regularized Ridge) to discriminate between CDI and PP. The predictive model was developed in the final cohort of 24 pediatric patients with CDI or PP, after exclusion of three NDI cases as outlined in the Methods section.

The predictor variables retained for modeling were stimulated copeptin levels, peak serum sodium, peak serum osmolality, test duration (minutes), and tolerability score (3-point Likert scale). No missing data were present. Continuous predictors were standardized (z-score transformation) to ensure scale comparability and to optimize the stability of the Ridge penalty. The binary outcome was final diagnosis (1 = CDI, 0 = PP). Baseline sodium, osmolality, and copeptin were excluded to avoid redundancy and to ensure that the model focused specifically on stimulus-induced diagnostic signals.

#### 3.3.1. Full-Sample Performance

**Figure 2:**
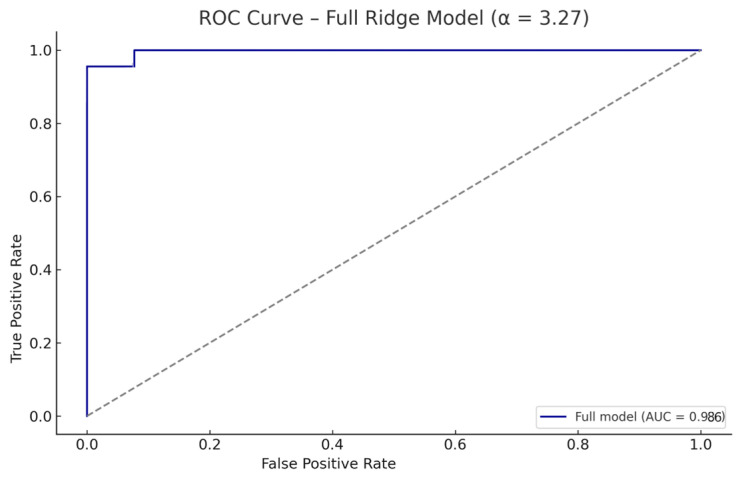
ROC Curve—Ridge logistic regression model (α = 3.27). The model achieved near-perfect discrimination between CDI and PP in the full dataset (n = 24).

**Figure 3:**
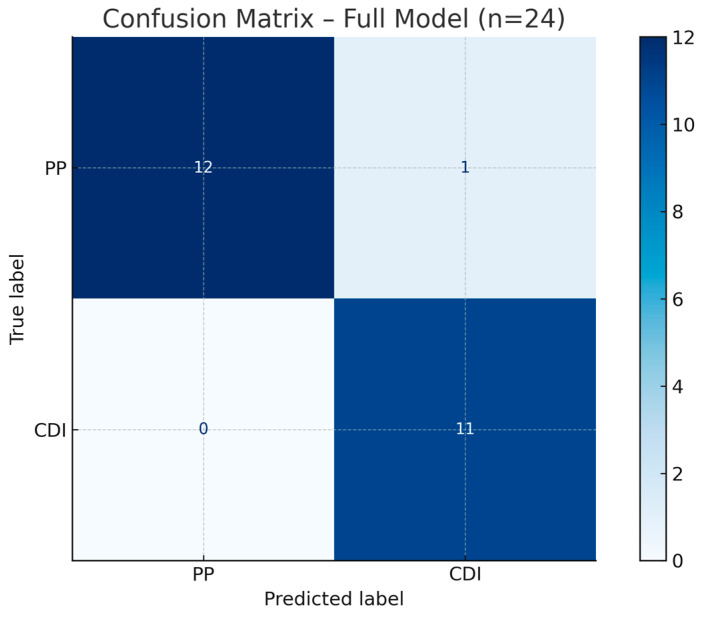
Confusion Matrix—Ridge logistic regression model. All CDI cases were correctly identified (sensitivity 100%), while one PP case was misclassified (specificity: 92.3%).

Ridge regularization was selected over Lasso due to the relatively small sample size and potential multicollinearity among predictors, with the goal of retaining all clinically relevant variables while minimizing overfitting. The Ridge model was trained using a fixed regularization parameter α = 3.27 (C = 0.305), based on prior calibration. When fit on the full dataset, the model demonstrated excellent discriminative capacity, with an area under the ROC curve (AUC) of 0.986 (Figure 2), an overall accuracy of 95.8%, 100% sensitivity for CDI, and 92.3% specificity for PP. Only one patient with PP was misclassified as CDI (Figure 3), likely due to a borderline copeptin response (5.9–6.3 pmol/L) and moderate test discomfort. This AUC reflects in-sample performance and is expectedly optimistic relative to cross-validated estimates; the internally validated AUC is reported in Section 3.3.2.

#### 3.3.2. Internal Validation via Stratified Cross-Validation

To estimate the model’s generalizability and avoid overfitting, we applied a 5-fold stratified cross-validation procedure using the same regularization parameter (α = 3.27) to obtain an unseen-data performance estimate. This approach ensured balanced class representation in all folds and preserved the proportions of CDI (n = 11) and PP (n = 13) across the validation sets.

**Figure 4:**
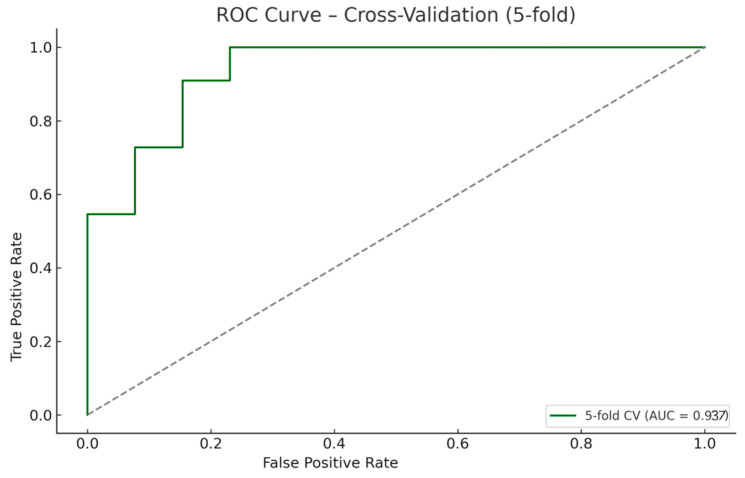
ROC Curve—cross-validation (5-fold). The model maintained high discriminatory performance (mean AUC = 0.937), confirming internal validity across folds.

The model achieved a mean cross-validated AUC of 0.937 (Figure 4), with a cross-validated accuracy of 83.3%, sensitivity of 81.8% for CDI, and specificity of 84.6% for PP. These results confirm that the Ridge model retained strong diagnostic performance in unseen data subsets despite the limited cohort size.

**Figure 5:**
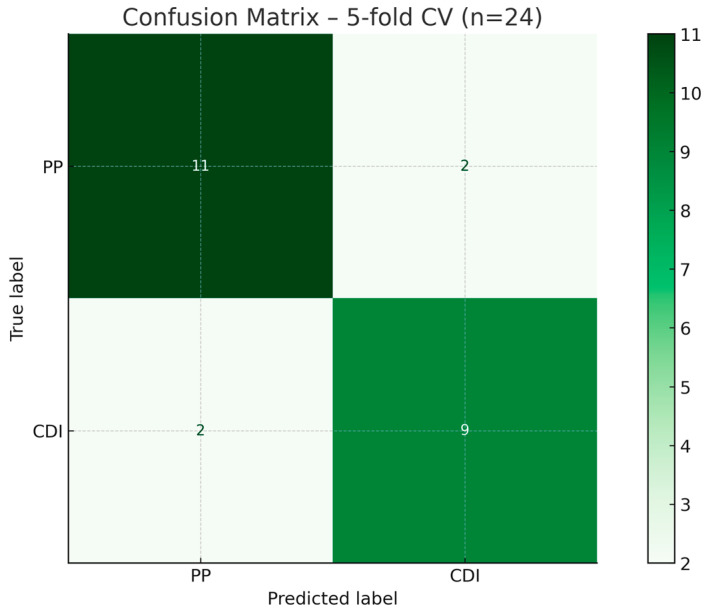
Confusion Matrix—5-fold cross-validation. Aggregated confusion matrix from cross-validated predictions shows two CDI cases that were misclassified as PP, and two PP cases misclassified as CDI, reflecting minor performance drop in internal validation (accuracy: 83.3%).

The confusion matrix from cross-validation (Figure 5) revealed two misclassified CDI cases (false negatives) and two misclassified PP cases (false positives), likely reflecting borderline biomarker responses or intermediate tolerability scores.

To confirm the robustness of the selected α, we performed sensitivity analyses across a broad range of regularization strengths (10^−2^–10^2^). The model’s performance remained stable, with mean AUC consistently between 0.93 and 0.97 for α values within the optimal interval, and α = 3.27 falling squarely in this plateau (Appendix A—Figure A1, Figure A2 and Figure A3). Both randomized folds (Figure A1 and Figure A2) and deterministic non-shuffled folds (Figure A3) demonstrated stable calibration, excluding the possibility that α selection was an artifact of data partitioning.

To assess the relative contribution of each parameter in distinguishing CDI from PP, we applied the same Ridge logistic regression model (Table 3), using the full cohort of 24 pediatric patients (CDI: n = 11; PP: n = 13). Prior to modeling, all continuous predictors were standardized using z-score transformation. The optimized regularization parameter (α = 3.27) was chosen to minimize overfitting while preserving interpretability.

**Figure 6:**
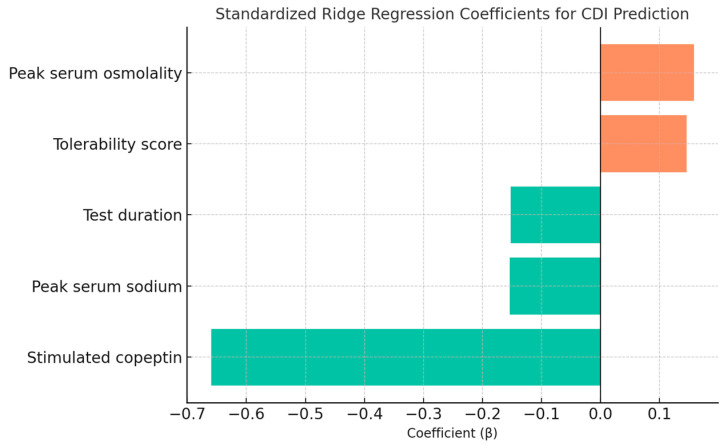
Standardized Ridge regression coefficients for CDI prediction. The plot highlights the dominant role of stimulated copeptin and the relatively minor contributions of serum osmolality, sodium, test duration, and tolerability score.

The model demonstrated that stimulated copeptin concentration was the most influential predictor in differentiating central diabetes insipidus (CDI) from primary polydipsia (PP), with a strong negative coefficient, as shown in Table 3 and Figure 6. This reflects the pathophysiological absence of vasopressin release in CDI and confirms the biomarker’s discriminative value after hypertonic saline infusion.

Among the clinical parameters, serum osmolality at peak stimulation and test tolerability score showed modest positive associations with CDI diagnosis (Table 3). However, test duration and peak serum sodium were weakly negative predictors (Table 3). These findings underscore the central role of copeptin as a robust biomarker, while also supporting the complementary diagnostic value of physiological and clinical tolerability parameters in pediatric polyuria–polydipsia syndrome.

The model intercept was –0.083, reflecting the baseline log-odds of a CDI diagnosis in the absence of predictor effects. All variables were z-score standardized prior to model fitting to ensure comparability of coefficients and to facilitate regularization. The small magnitude of the intercept suggests well-balanced class distribution and appropriate model centering.

These results suggest that a multimodal approach combining stimulated copeptin levels with sodium/osmolality dynamics and tolerability assessment can significantly enhance the diagnostic accuracy for CDI versus PP in pediatric patients, especially when classic criteria yield borderline or inconclusive results.

Although no independent pediatric cohort was available for external validation due to the rarity of PPS, the 5-fold stratified cross-validation approach provides robust internal evidence for the model’s generalizability. Future multicenter studies with larger sample sizes are needed to externally validate and refine this algorithm.

## 4. Discussion

Pediatric polyuria–polydipsia syndrome (PPS) sits at a difficult intersection of biology, feasibility, and patient comfort: clinicians must decide quickly between central diabetes insipidus (CDI) and primary polydipsia (PP) using tools that are accurate, tolerable, and practical at the bedside. Our study addresses this decision point by shifting from single-threshold interpretations of copeptin to an integrative framework that combines biomarker dynamics with simple clinical readouts, using a standardized osmotic stimulation test. The results of our study constitute the first step in obtaining a valuable clinical tool based on a statistical penalized regression model.

While copeptin has already been validated as a robust biomarker for distinguishing CDI from PP, most studies have relied on single cut-off values [31,46,47,49,55,56,57,58,59,60,61]. This approach, although simple, is limited by interindividual variability, incomplete osmotic stimulation and the presence of borderline cases, that can undermine diagnostic certainty. Our study moves beyond cut-offs and demonstrates the feasibility of a multimodal algorithm that integrates biomarker dynamics with clinical parameters, offering a more nuanced decision-making tool. The main findings reinforce the central pathophysiological signal provided by stimulated copeptin while showing that small, clinically meaningful additions—peak osmolality, peak sodium, test duration, and a brief tolerability score—stabilize classification in exactly those “gray-zone” scenarios that are hardest for clinicians. In line with expectations, stimulated copeptin emerged as the dominant predictor (β = –0.660), reflecting its central pathophysiological role in CDI, while sodium/osmolality dynamics and tolerability contributed to a more resilient diagnostic profile. Basal parameters were excluded from the model to avoid redundant physiological information and to focus on the dynamic response elicited by osmotic stimulation, which more accurately reflects hypothalamic–pituitary integrity. Similar strategies have been applied in pediatrics, where multimodal machine learning (ML) has been used for differential diagnosis of Kawasaki disease versus other febrile illnesses [62], triage in resource-limited settings [63], or prognostic modeling in pediatric brain tumors [64]. These precedents reinforce that composite algorithms mirror clinical reasoning more closely than isolated thresholds, and they underline the translational potential of our approach for PPS.

Methodologically, our choice of ridge logistic regression was deliberate. Rare pediatric cohorts magnify variance, inflate coefficients, and challenge reproducibility when conventional multivariable models are fitted without constraints. Therefore, using a traditional multivariable logistic regression is prone to overfitting in small samples, which are unavoidable in rare pediatric conditions such as PPS. Ridge penalization shrinks coefficients just enough to dampen instability while preserving all clinically relevant predictors in the model, which represents a real advantage when clinicians need the full clinical picture for decision making. By applying Ridge regression with stratified cross-validation, we minimized this risk and generated a model that is not only interpretable but also robust to variability. Penalized regression approaches are increasingly used in rare pediatric disorders, including neurodegenerative syndromes [43], SIADH [44], and sepsis-associated kidney injury [65]. Methodological work also shows that Ridge regression provides more stable and clinically useful predictions than unpenalized or Lasso models when sample sizes are small and predictors are correlated [42,66]. While more complex machine learning classifiers are often applied in biomedical research, evidence suggests that in small medical datasets penalized regression models such as Ridge logistic regression can provide equally strong or even superior predictive performance, with the added advantage of interpretability [67]. This evidence supports our choice to prioritize penalized regression with internal cross-validation, rather than more opaque classifiers, ensuring both robustness and clinical interpretability.

The performance pattern also speaks about generalizability. When trained on the full dataset, the model showed excellent discrimination (AUC 0.986), inevitably overestimating accuracy. To overcome this issue, we performed, in addition, internal validation, that provides a more realistic benchmark for unseen patients, yielding a mean AUC of 0.937 with balanced sensitivity and specificity. This aspect indicates that performance persists on unseen splits of the data and is unlikely to be an artifact of a single partition.

Another aspect that should be clarified is regarding the apparent discrepancy between the near-perfect AUC of hypertonic saline–stimulated copeptin reported in our previous work (AUC = 1.0 in a univariate setting [36]) and the present Ridge regression model, which is totally expected and reflects methodological differences. Furthermore, sensitivity analyses across a wide range of regularization strengths showed a stable AUC plateau (see Appendix A), placing the selected α squarely within a region of robustness and reducing the risk that our estimate rests on a narrow tuning choice. Taken together, these results argue for a model that travels better to new patients than an unpenalized fit would.

Interpreting the coefficients offers clinically intuitive signals. The negative weight for stimulated copeptin aligns with absent or blunted AVP release in CDI. The small but consistent contributions of peak osmolality (β = +0.158) and tolerability (β = +0.146) emphasize that clinical robustness stems from combining physiological readouts with patient-centered measures, even when their individual effects are modest. Our contribution is not to surpass copeptin’s biological performance, but to demonstrate that a multimodal, penalized regression framework preserves diagnostic accuracy while integrating tolerability and sodium/osmolality dynamics—parameters that are clinically relevant when copeptin results are borderline or equivocal. This framework also provides a safeguard against overoptimistic results, which is a frequent pitfall in rare pediatric diseases where sample sizes are small.

Internal validation with stratified 5-fold cross-validation is a widely accepted standard in pediatric ML research, used, for example, in models predicting ECMO-related brain injury [68] or in pediatric autism diagnosis using Ridge regression [69]. Our results parallel these frameworks, showing that even in rare pediatric cohorts, cross-validation ensures that diagnostic models are not artifacts of small-sample variability but reflect clinically translatable performance.

**Figure 7:**
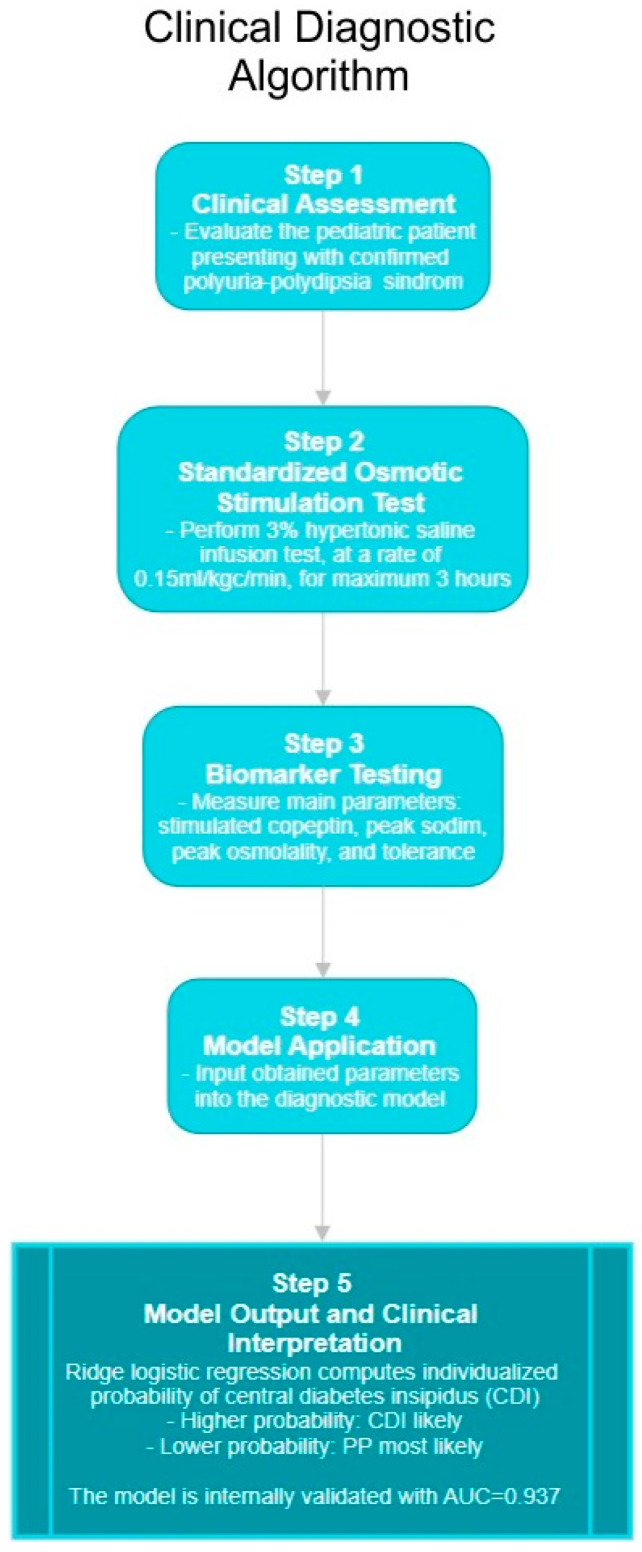
Clinical Diagnostic Algorithm for Pediatric Polyuria–Polydipsia Syndrome. Schematic representation of the proposed multimodal diagnostic workflow integrating clinical evaluation, standardized hypertonic saline stimulation, biomarker assessment (stimulated copeptin, peak sodium, peak osmolality, and tolerability score), and model-based interpretation.

In clinical aspects, our findings suggest that copeptin should not be interpreted in isolation but in combination with sodium/osmolality dynamics and tolerability measures. One physiologic aspect is the fact that beyond osmotic regulation, copeptin can also rise in response to non-osmotic stressors (e.g., glucagon or insulin tolerance tests [70,71]) or even in multisystemic inflammatory responses [16,72]. But in such settings, AVP/copeptin appears to play only a limited role, underscoring the value of osmotic stimulation when using copeptin diagnostically. For example, borderline copeptin values around the diagnostic threshold (<6.5 pmol/L) can be reclassified more reliably when integrated into a multimodal model. This algorithmic approach reduces the risk of misclassification and allows pediatric endocrinologists to provide more confident guidance to families. Similar to ML-based calculators already tested in pediatric sepsis and febrile illnesses [62,63,65], our model could be implemented as a clinical decision-support tool: inputting a child’s copeptin, sodium, osmolality, and tolerability score would yield an individualized probability of CDI versus PP. This translation into a bedside application underlines the clinical relevance of our work, particularly in pediatrics, where rapid and accurate differentiation directly impacts treatment choice, fluid management, and long-term outcomes. To facilitate clinical translation of these findings, a simplified step-by-step diagnostic algorithm is illustrated in Figure 7, summarizing the sequential workflow from patient assessment to model-based interpretation.

Feasibility and safety deserve equal emphasis in pediatrics, and our protocol performed well on both counts. The hypertonic saline test was safe in our cohort, with no severe adverse events and only transient, self-limiting symptoms. Tolerability strongly correlated with test duration, supporting its inclusion as a predictor. This pragmatic insight is important for clinical workflows: shorter tests not only reduce discomfort but also conserve resources, making the hypertonic saline–copeptin protocol more feasible than the water deprivation test [36]. Although our tolerability score was pragmatic and limited, similar to other pediatric ML studies where custom clinical scales were employed [63], it captured clinically relevant differences. Future multicenter trials should validate such tools against standardized pediatric symptom scales.

Several obstacles shaped the study and indicated the resources needed going forward. First, the rarity of pediatric PPS determined a small sample size and the lack of an independent validation cohort. Nevertheless, stratified cross-validation provided robust internal evidence of generalizability. Similar pediatric ML studies—whether in cardiology, nephrology, or neurodevelopmental disorders—have demonstrated that internal validation is a meaningful step prior to multicenter confirmation [42,64,68,69,73,74,75]. Future research should extend our algorithm to multicenter cohorts, potentially adding complementary metrics such as thirst perception scales, genetic markers of AVP signaling, or imaging features. In future research particular attention should be focused on laboratory harmonization (minor inter-assay differences in copeptin thresholds are plausible [59]), explicit calibration (relevant statistical methods [76]), and clinical utility assessment [77]. Importantly, the methodological framework we propose—penalized regression with multimodal integration—can serve as a blueprint for other rare pediatric diseases where sample sizes are small but diagnostic accuracy is critical.

In the longer term, validated multimodal algorithms such as this could inform clinical guidelines and be implemented as digital decision-support systems, bridging the gap between biomarker science and routine pediatric endocrinology.

## 5. Conclusions

This study demonstrates that integrating stimulated copeptin with clinical parameters into a multimodal Ridge regression model provides a clinically robust approach for differentiating central diabetes insipidus from primary polydipsia in children. By moving beyond single biomarker thresholds, our work highlights the value of penalized regression and internal cross-validation as essential tools in rare pediatric conditions, where small sample sizes limit traditional validation strategies. The model proved safe, feasible, and interpretable, and its translational potential lies in developing clinical decision-support applications that can assist pediatric endocrinologists in real time. While external validation in larger, multicenter cohorts remains necessary, our findings offer a methodological and practical framework that could inform future diagnostic protocols for polyuria–polydipsia syndrome and related disorders.

## Figures and Tables

**Table 1:** Comparative distribution of clinical and biochemical variables between patients with central diabetes insipidus (CDI) and those with primary polydipsia (PP).

Variable	PP (n = 13)	CDI (n = 11)	*p*-Value (Mann–Whitney U)
Age (years)	14.58 (9.67–16.58)	7.92 (4.83–10.42)	-
Weight (kg)	41.0 (35.0–55.0)	26.0 (20.0–30.0)	-
Stimulated copeptin (pmol/L)	19.0 (14.0–23.4)	4.92 (3.1–6.8)	<0.001
Basal copeptin (pmol/L)	4.05 (3.23–5.20)	1.86 (1.23–2.65)	<0.001
Baseline sodium (mmol/L)	141.0 (138.0–142.0)	144.0(142.0–146.0)	0.0807
Maximum sodium (mmol/L)	150.0 (149.1–150.0)	150.0 (150.0–151.0)	0.3294
Baseline osmolality (mOsm/kg)	283.0 (276.6–286.0)	283.0 (280.0–285.0)	0.5615
Maximum osmolality (mOsm/kg)	301.0 (300.0–303.0)	302.0 (301.0–304.0)	0.5998
Test duration (min)	180.0 (180.0–180.0)	120.0 (120.0–150.0)	0.1074
Tolerance (1–3) ^1^	1.0 (1.0–1.0)	2.0 (1.0–2.0)	0.0329

^1^ simple score 1–3: 1 = good, 2 = moderate, 3 = poor. Values are expressed as median (IQR).

**Table 2:** Variance Inflation Factors (VIFs) for candidate predictors included in the multivariable model.

Variable	VIF ^1^
Basal copeptin	3.44
Stimulated copeptin	3.53
Baseline sodium	1.62
Peak sodium	1.89
Baseline osmolality	2.42
Peak osmolality	2.86
Test duration	1.71
Tolerability score	1.75

^1^ All variables showed VIF < 5. The intercept (constant term) was also evaluated and yielded a VIF of 1.06, as expected for non-informative predictors.

**Table 3:** Ridge regression coefficients for the prediction of central diabetes insipidus (CDI) versus primary polydipsia (PP).

Predictor	Coefficient (β)
Intercept	–0.083
Stimulated copeptin (standardized)	–0.660
Peak serum sodium (standardized)	–0.154
Peak serum osmolality (standardized)	+0.158
Test duration (standardized)	–0.152
Tolerability score (standardized)	+0.146

## Data Availability

Personal medical data are publicly unavailable due to privacy or ethical restrictions, being obtained from the medical record of the patient admitted into “St. John” Emergency Clinical Hospital for Children in Galati, Romania. De-identified data supporting the findings are available from the first author upon reasonable request and with institutional approval.

## Abbreviations

The following abbreviations are used in this manuscript:

AVP	Arginine Vasopressin
AUC	Area Under the Curve
CDI	Central Diabetes Insipidus
EDTA	Ethylenediaminetetraacetic Acid
FIA	Fluorescence Immunoassay
GDPR	General Data Protection Regulation
NDI	Nephrogenic Diabetes Insipidus
PP	Primary Polydipsia
PPS	Polyuria–Polydipsia Syndrome
ROC	Receiver Operating Characteristic
STARD	Standards for Reporting Diagnostic Accuracy Studies
TRPV1/4	Transient Receptor Potential Vanilloid 1/4
VIF	Variance Inflation Factor
WDT	Water Deprivation Test

## Appendix A. Supplementary Analyses of Model Calibration and Regularization Stability

To further examine the robustness of the Ridge regression model, we conducted sensitivity analyses across a wide range of regularization strengths (α values). These figures illustrate that the model’s diagnostic performance remained stable regardless of the exact α, confirming that the chosen value (α = 3.27) lies within a broad plateau of optimal calibration.

These supplementary analyses further support that the Ridge model’s excellent diagnostic performance (AUC > 0.93 across all tested values) is not an artifact of overfitting or of arbitrary parameter tuning. This stability provides additional reassurance that the algorithm can be generalized beyond the present dataset, although external validation in larger pediatric cohorts remains necessary.
